# Extraction Optimization, UHPLC-Triple-TOF-MS/MS Analysis and Antioxidant Activity of Ceramides from Sea Red Rice Bran

**DOI:** 10.3390/foods11101399

**Published:** 2022-05-12

**Authors:** Gang Wang, Xue-Jing Jia, Bing-Bing Song, Rui Li, Xiao-Fei Liu, Jian-Ping Chen, Sai-Yi Zhong, Hong-Kai Zhou

**Affiliations:** 1 College of Food Science and Technology, Guangdong Ocean University, Guangdong Provincial Key Laboratory of Aquatic Product Processing and Safety, Guangdong Province Engineering Laboratory for Marine Biological Products, Guangdong Provincial Engineering Technology Research Center of Seafood, Guangdong Provincial Science and Technology Innovation Center for Subtropical Fruit and Vegetable Processing, Zhanjiang 524088, China; wg455149527@163.com (G.W.); jiaxj@gdou.edu.cn (X.-J.J.); 15891793858@163.com (B.-B.S.); liruihn@163.com (R.L.); liuxf169@126.com (X.-F.L.); cjp516555989@gdou.edu.cn (J.-P.C.); 2 Shenzhen Research Institute, Guangdong Ocean University, Shenzhen 518108, China; 3 Collaborative Innovation Center of Seafood Deep Processing, Dalian Polytechnic University, Dalian 116034, China; 4 Coastal Agricultural College, Guangdong Ocean University, Zhanjiang 524088, China; zhouhk@gdou.edu.cn

**Keywords:** ultrasonic-assisted extraction, response surface methodology, structure, antioxidant activity, ceramide, sea red rice

## Abstract

As a new type of salt-tolerant rice, sea red rice contains more minerals, proteins, and lipid compounds, and, in particular, its by-product rice bran may be used to replace other commercial rice brans as the main source of ceramides (Cers). However, the extraction rate of Cers is generally low, and it is crucial to seek an efficient extraction method. This study optimized the ultrasonic-assisted extraction of Cers from sea red rice bran using response surface methodology (RSM) and obtained a Cers yield of 12.54% under optimal conditions involving an extraction temperature of 46 °C, an extraction time of 46 min, and a material–to-liquid ratio of 5 g/mL. The Cers content in sea red rice bran was preliminarily analyzed using thin-layer chromatography, and the Cers content was determined via UHPLC-Triple-TOF-MS/MS after purification and separation using silica column chromatography. Forty-six different types of Cers were identified in sea red rice bran, of which Cer 18:0/24:0 (2OH), Cer 18:0/26:0, Cer 18:0/26:0 (2OH), and Cer 18:0/24:0 accounted for 23.66%, 17.54%, 14.91%, and 11.96%. Most of the Cers structures were mainly composed of sphingadienine. A biological activity assay indicated that Cers extracted from sea red rice bran had significant antioxidant and anti-aging properties. These findings indicate that the extracted Cers show great potential for applications in the cosmetic and pharmaceutical industries.

## 1. Introduction

Sea red rice, a salt-tolerant rice that grows in the most southern coastal areas of China, generates purple-red bran. Due to the special nature of the environment that it grows in, pesticides are not required. This is a new, recently discovered rice seed that has not bee not mass-used [1,2]. It reportedly contains high amounts of minerals, proteins, and lipid compounds, as well as biologically active substances. Rice bran, which is a by-product of the whitening process of shelled brown rice, amounts to approximately 12% of the total rice milled. Currently, red sea rice bran is not being fully utilized. For example, it is rarely used as animal feed, due to its special color and slightly salty taste.

According to our estimates, thousands of kilograms of sea red rice bran are discarded as garbage every year, which is a great waste of resources. Therefore, maximizing the efficacy of these by-products may enhance economic cycles and environmental protection. Rice bran contains polyphenols (gallic acid and protocatechuic acid), phospholipids, vitamins (including γ-oryzanol, vitamin E, and δ-tocopherol), and flavonoids (kaempferol), as well as ceramide (Cer), nicotinamide, minerals, and proteins, which are known to possess antioxidant, anti-inflammatory, anti-aging, and immune regulatory properties [3,4,5]. 

Furthermore, phytoceramide, a relatively rare substance in rice bran, is often used in cosmetics and medical research due to its better antioxidant capabilities and skin affinity. The types and stuctures of Cers found in sea red rice bran remain largely unexplored and unreported. Therefore, it may have high research value and exploratory significance [6].

The extraction of bioactive compounds from plant tissues may be accomplished via one of several methods. The most frequently used methods include solvent maceration and soxhlet extraction, both requiring organic solvents. In addition to conventional extraction techniques, Cer may also be extracted from wool fiber amides via supercritical fluid extraction (SFE), which, however, requires a variety of equipment and is costly. Therefore, other assisted extraction techniques, such as enzyme-assisted extraction (EAE) and ultrasonic-assisted extraction (UAE), have gained importance [7,8,9]. 

The highly effective UAE technique (which requires less solvent, displays a significant thermal effect, improves the extraction time, is cost-efficient, and involves easy handling) has been identified as a promising alternative to conventional extraction. This method helps circumvent the issue of reduced enzyme activity caused under EAE conditions, thereby, increasing the utilization rate of many plant materials. Therefore, it is considered a more useful extraction method and has been used in the recovery of anthocyanins and phenols from mulberry pulp, antioxidants from mandarin and lime peels, and polysaccharides from dragon fruit [10,11].

Cers are specific sphingolipid metabolites that have been identified as lipid compounds, which are mostly composed of sphingoid bases esterified into long-chain FAs or very-long-chain FAs (>20 carbon chains), such as sphingomyelin and glycosphingolipids. Cers play an important physiological role by facilitating activities, such as the maintenance of cell membrane stability and prevention of skin moisture loss, among others [12]. To date, 18 different Cer classes have been reportedly identified in human skin [13]. The unique properties of Cers enable these to be widely used in the cosmetic industry with particular reference to the preparation of skin creams and shampoos. 

Extraction from animal tissues (such as bovine brains, pigskins, and eggs) is possible but presents certain risks and limitations. Conversely, plant Cers (which mainly exist in glycosylated, acylated, or fatty acid forms containing multiple hydroxyl groups) show good biological activity. Although Cers are the most widespread sphingolipids in plants, free Cers make up only a minor fraction of sphingolipids. They occur naturally in many grains, legumes, and dietary sources, mostly in the form of derivatives, such as wheat, rice, and konjac [14]. The types and structures of Cers present in rice bran are reportedly closer to those found in human skin [15]. 

The usefulness of plant Cers for improving oxidative skin damage has attracted much attention due to their higher safety profile, which is linked to being mostly isolated from dietary sources. Several studies have revealed the benefits of using orally ingested plant Cers for purposes, such as skin hydration and anti-aging in both animal and healthy human subjects [6,16]. The basic exploration of Cers in sea red rice bran to evaluate its potential industrial application is needed. The purpose of this study was to further verify the content and type of Cers in sea red rice bran and its antioxidant activity on the basis of improving the yield of Cers in sea red rice bran. 

Therefore, in this study, we aimed to improve the Cer yield by using an ultrasonic-assisted method and optimizing the extraction temperature, extraction time, and material-to-liquid ratio using the Box–Behnken design (BBD). The potential for expanding the yield of extracted Cers was analyzed using thin-layer chromatography (TLC) and silica column chromatography, following which the types and contents of Cers were determined via advanced analytical tools using UHPLC-Triple-TOF-MS/MS, and the components of Cers were compared. 

Finally, the anti-aging and antioxidant activities of Cers in sea red rice bran were evaluated. This study laid a foundation for the development and comprehensive utilization of Cers in sea red rice bran. 

## 2. Materials and Methods

### 2.1. Materials and Reagents

Sea red rice is grown in Zhanjiang City, Guangdong Province, China. Sea red rice bran, provided by Professor Zhou of the Agricultural College of Guangdong Ocean University, was stored in a refrigerator at 4 °C before being used for the experiment. Standard Cer (from commercial rice bran purity 99%) was purchased from Beijing Lianchuang Biological Research Institute (Beijing, China). 

Additionally, 95% ethanol, ethyl acetate, petroleum ether, chloroform, anhydrous copper sulfate, phosphoric acid solution, benzoyl chloride, anhydrous pyridine, (all of which were analytically pure), as well as Cer (d18:1/15:0) as an internal standard, cellulase, tyrosinase, and elastase were purchased from Sigma-Aldrich (Dorset, UK). Silica gel 60 (0.063–0.20 mm) and TLC (Silica gel 60, HSGF254, 5 cm × 10 cm) were purchased from Qingdao Ocean Chemical (Qingdao, China). DPPH and the hydroxyl radical kit were purchased from Zhanjiang Keming Biological Reagent Co., LTD. (Zhanjiang, China). Deionized water was used in all experiments.

### 2.2. Extraction of Cers

Sea red rice bran was cleaned and crushed using a 140-mesh sieve (aperture 0.105 mm, Qingdao Ocean Chemical, Qingdao, China). Then, 50 g of rice bran (accurate to 0.01 g) was accurately weighed into a 500 mL beaker. Next, 95% ethanol was added at a volume greater than about five times the sample volume, and the resulting solution was left to stand for a certain time to ensure that the rice bran was completely soaked by the solvent. 

Subsequently, the ultrasonic processor (IRM Technology GmbH, DTH030, Hannover, Germany) was turned and the ultrasonic frequency was set to 40 KHZ. The extraction temperature was set to a predetermined value after about 50 °C. Then, the beaker was placed in the ultrasonic processor. The extraction was performed at a power of 360 W for some time. The extract in the beaker was removed and left to stand for approximately 5 min and was then filtered under heat and reduced pressure. 

The filtered extract was concentrated under reduced pressure in a rotary evaporator (EYELA, N-1300V-WB, Tokyo, Japan); petroleum ether was then added to the partition funnel at an amount greater than about twice the volume of the extract, and the concentrate was separated from ethanol via extracting and eluting to filter out maximum amount of residual ethanol solution. Finally, the extract was dried in a vacuum drying box (IRM Technology GmbH, VD110, Hannover, Germany) at 60–70 °C for 3–4 h until there was no petroleum ether and residual ethanol. By ensuring that the weight remained constant, the Cers extract was finally obtained [17]; its yield was estimated using the following formula:

The yield of Cers extract (%) = [crude extract (g)/rice bran powder (g)] × 100%

### 2.3. Procedures for Conventional Extraction and EAE

Sea red rice bran was placed in a 500 mL round-bottomed flask to which 95% ethanol was added at a volume greater than about five times the sample volume; the resulting solution was condensed and refluxed for extraction by connecting a condenser tube. The other steps were the same as those described for the UAE procedure. Following extraction, the resulting solution was filtered with filter paper, and the extract in the beaker was taken out and left to stand for approximately 5 min. The extract was filtered and concentrated under reduced pressure, and then petroleum ether was added to the partition funnel at a volume that was approximately twice the volume of the extract; finally, the resulting product was extracted and dried.

Next, the solute was treated with 1% cellulase (according to the solute volume and mass fraction) and extracted at 42 °C and pH 4.5. The other steps were similar to those used for the UAE procedure. After enzymatic hydrolysis, the extract in the beaker was taken out, left to stand for approximately 5 min, and then heated and filtered under reduced pressure. Subsequently, petroleum ether was added to the partition funnel at a volume that was approximately twice the volume of the extract; finally, the resulting product was extracted and dried.

### 2.4. Single-Factor Experiment

In this experiment, the organic solvent ultrasonic-assisted method combined with the RSM method was used, with the Cers yield as the index. Before the RSM was performed, the selection range of RSM was determined using the single-factor method. The effects of changes in the following four single factors on the Cers yield were estimated: the material-to-liquid ratio (1:3, 1:4, 1:5, 1:6; and 1:7); extraction solvent (95% ethanol, petroleum ether, ethyl acetate, and chloroform); extraction time (25, 35, 45, 55, and 65 min); and extraction temperature (40, 50, 60, 70, and 80 °C). Each experiment was executed in triplicate.

### 2.5. Optimization of Ultrasonic-Assisted Extraction Via Box–Behnken Design

Based on the results of single-factor extraction in the preliminary experiment, three factors influencing extraction yield, namely, the extraction temperature (A), extraction time (B), and material-to-liquid ratio (C), were selected as independent variables based on the experimental design principle of the Box–Behnken software. Three levels, low, medium, and high, which were coded as −1, 0, and 1, respectively, were assigned to each factor. Finally, using the Cers yield as the response value, the UAE was used to optimize the extraction. The experimental results were analyzed via analysis of variance (ANOVA). Design-Expert Software was used for the experimental design, regression, and image data analysis of the obtained results.

### 2.6. Thin-Layer Chromatography (TLC)

TLC was used to perform a qualitative analysis of the extract to identify the presence of amides [18]. A silica gel thin-layer plate (5 × 10 cm) was used as the stationary phase. Before use, the silica gel plate was activated in an oven at 100 °C–105 °C for 0.5 h, and the sample was dissolved in methanol (analytical grade) with a developing agent (chloroform: methanol: acetic acid = 19:0.9:0.1 (*v*/*v*/*v*)). 

A 0.5 × 100 mm capillary was used for sampling, following which the thin-layer plate was developed in the developing agent and then dried for 10 min. Next, the silica gel plate was immersed in 10% copper sulfate-phosphoric acid solution, ensuring that the chromogenic agent was completely submerged. After 3 min, it was removed and dried for 10 min, then dried in an oven at 130 °C for a further 20 min, removed, and examined for color change.

### 2.7. Purification of Cers

Cer extracts were separated and purified using a silica gel column (normal, 20 × 300 mm) purchased from Qingdao Ocean Chemical chromatography. Eluents, 1 (ethyl acetate: petroleum ether = 6:4), 2 (ethyl acetate: petroleum ether = 1:1), and 3 (acetone: petroleum ether = 6:4) were used as elution agents to develop an elution gradient. Each elution volume was 250 mL, and the elution fractions were collected and divided by placing them in 500 mL conical bottles. 

The elution flow rate was 1 d/s. Samples were separated and purified using silica gel column chromatography thrice, following which, the gradient eluent was collected and detected using TLC. The eluent containing Cers was concentrated with a rotary evaporator and dried for 2 h in a vacuum drying box at 60 °C to obtain a small amount of liquid. Mass-grade methanol, used as the solvent, was slowly added along the cup wall at a ratio of 1:2, recrystallized, and purified at 4 °C to obtain white powdery particles [19].

### 2.8. Structural Characterization of Cers by UHPLC-Triple-TOF-MS/MS

Cers profiling was accomplished via a UPLC 30A system (Shimadzu Corporation, Kyoto, Japan) coupled with a Sciex Triple-TOF^®®^ 6600 (AB Sciex, Concord, ON, Canada). Briefly, Cers extracts were separated using a Phenomenex Kinetex C18 column (100 × 2.1 mm, 2.6 μm) and a Phenomenex Security Guard precolumn of the same material (Phenomenex, Torrance, CA, USA). The injection volume for positive and negative ESI modes were all 1 μL, respectively. The column temperature was maintained at 60 °C, and the flow rate at 0.4 mL/min. 

A gradient elution was started with 20% solvent B (IPA/ACN = 5:1, *v*/*v*; 5 mM ammonium acetate) and 80% solvent A (water/MeOH/ACN = 1:1:1, *v*/*v*/*v*; 5 mM ammonium acetate) for 0.5 min, increased to 40% B from 0.5 to 1.5 min, to 60% B from 1.5 to 3 min, to 98% B from 3 to 13 min, decreased to 20% B from 13.1 min, and maintained at 20% B from 13.1 to 17 min, for a total run time of 17 min. Chromatographic and mass spectrometry data were acquired using Applied Biosystems 1.6 software, in both ESI+ and ESI− modes with the information-dependent acquisition (IDA). 

The MS parameters were as follows: declustering potential set to 80 V (+) and −80 V (−); collision energy to 10 V (+) and −30 V (−); ion spray voltage to 5500 V (+) and −4500 V (−), and mass range to *m*/*z* 100 to 1600. The pressure of the ion source gas was set to 1 and ion source gas to 2; curtain gas to 50, 50, and 35 psi, respectively; and the interface heater temperature to 600 °C [20,21]. 

### 2.9. Software Data Analysis and Cers Identification

Freely available MS-DIAL, version 4.00 (http://prime.psc.riken.jp/Metabolomics_Software/MS-DIAL/index2.html, accessed on 1 February 2022), and commercially available software packages, PeakView, MasterView, MultiQuant (SCIEX), PubChem, and Phenol-Explorer, with relevant literature, were used for Cers profiling. All Cers species were identified via mass accuracy (precursor ion, <0.01 Da; fragment ion, <0.05 Da), isotopic pattern (difference, <10%), retention time tolerance (5 min), and MS accurate mass tolerance (0.01 Da). 

Following Cers identification, a Cers quantitative method was established, including the Cer name, retention time, and *m*/*z* using MasterView and PeakView, and this method was further applied for more in-depth quantitative analysis using MultiQuant (version 3.0.3). The contents of the identified Cer species were quantified by the selected internal standard (IS), while relative peak areas (analyte area/IS area) were used for quantification [20,21].

### 2.10. Antioxidant Activity Assays

#### 2.10.1. DPPH Radical Scavenging Rate Experiment

This experiment was based on a previously described method (Tessema et al., 2017), which used 1-diphenyl-2-picrylhydrazine (DPPH) radical of furfuryl Cers from rice husks, with slight modifications [22]. The sample was dissolved to produce 1 mg/mL in MASS methanol and diluted into different gradients of 0.2–1.0 mg/mL. Then, 3 mL samples were mixed with 1 mL of freshly prepared DPPH solution (0.2 mm of 95% ethanol solution) and shaken evenly. The mixture was kept away from light for 30 min, after which the sample absorbance was measured at 517 nm using a Thermo Arioskan Flash automatic microplate instrument (Thermo Corporation, Waltham, MA, USA). Vitamin C was used as a positive control. The standard solutions were selected and calibrated in the same range as the sample set. The DPPH radical scavenging capacity was calculated as follows:

$$\mathrm{DPPH}\;\mathrm{free}\;\mathrm{radical}\;\mathrm{scavenging}\;\mathrm{activity}\;(\%)=\left(1-\frac{A_{1}}{A_{0}}\right)\;\times \;100\%$$

where *A*_0_ is the absorbance of the blank control and *A*_1_ is the absorbance of the samples.

#### 2.10.2. Hydroxyl Radical Scavenging Rate Experiment

The hydroxyl radical scavenging activity of furfuryl Cers obtained from rice husk products and dissolved in 1 mg/mL of MASS methanol was monitored using a modified Fenton reaction [23]. The mixture was then diluted into differing gradients of 0.2–1.0 mg/mL. Next, the sample was mixed with 1 mL of ferrous sulfate, hydrogen peroxide, and salicylic acid. 

Subsequently, this mixture was allowed to react at 37 °C for 30 min, after which the sample absorbance at 510 nm was measured using a Thermo Arioskan Flash automatic microplate instrument (Thermo Corporation, Waltham, MA, USA). Vitamin C was used as the positive control. The standard solutions were selected and calibrated in the same range as the sample set. The hydroxyl radical scavenging capacity was calculated as follows:

$$\mathrm{Hydroxyl}\;\mathrm{free}\;\mathrm{radical}\;\mathrm{scavenging}\;\mathrm{activity}\;(\%)=\left(1-\frac{A_{1}}{A_{0}}\right)\;\times \;100\%$$

where *A*_0_ is the absorbance of the blank control and *A*_1_ is the absorbance of the samples.

### 2.11. Anti-Aging Activity Assay

#### 2.11.1. Tyrosinase Inhibition Test

The absorbance of dopaquinone, catalytically produced via L-DOPA (2.5 mM), was measured using a Thermo Arioskan Flash automatic microplate instrument (Thermo Corporation, Waltham, MA, USA) [24]. Tyrosinase (40 U/mL, PBS50 Mm) was added to the 96-well microtiter plate from left to right via a pipette and incubated at 37 °C for 10 min; following which, the substrate, L-DOPA, was added. Its absorbance at 475 nm was measured after 10 min. Kojic acid was used as a positive control. The formula used in the DPPH radical scavenging rate experiment was adopted.

#### 2.11.2. Elastase Inhibition Test

Based on previously described methods [25], the following procedures were performed: 10 μL of sample solution was added to 130 μL of 1.015 mmol/L reaction substrate, *N*-(methoxysuccinyl)-L-alanyl-L-alanyl, in a 96-well plate containing L-prolyl-L-valine-4-nitroaniline standard solution in 0.1 mol/L Tris-HCl buffer solution (pH 8.0), incubated at 25 °C for 5 min, treated with 15 μL elastase solution (0.5 U/mL), and incubated for a further 30 min at 25 °C; following which, the absorbance was measured at 410 nm using a Thermo Arioskan Flash automatic microplate instrument (Thermo Corporation, Waltham, MA, USA). The aqueous sample solution was replaced with deionized water, which was used as a reference solution, and its absorbance was measured. EGCG was used as a positive control.

### 2.12. Statistics and Analysis

Design-Expert 12.0.3 version (Stat-Ease, Minneapolis, MN, USA) was used to analyze the results of the RSM design. SPSS 2019 (SPSS Inc., Chicago, IL, USA) was used for other statistical analyses. All experiments were performed in triplicate. Variance analysis and Waller–Duncan multiple range tests were used to determine the significance of differences between the mean values.

## 3. Results

### 3.1. Single-Factor Experiment

**Figure 1:**
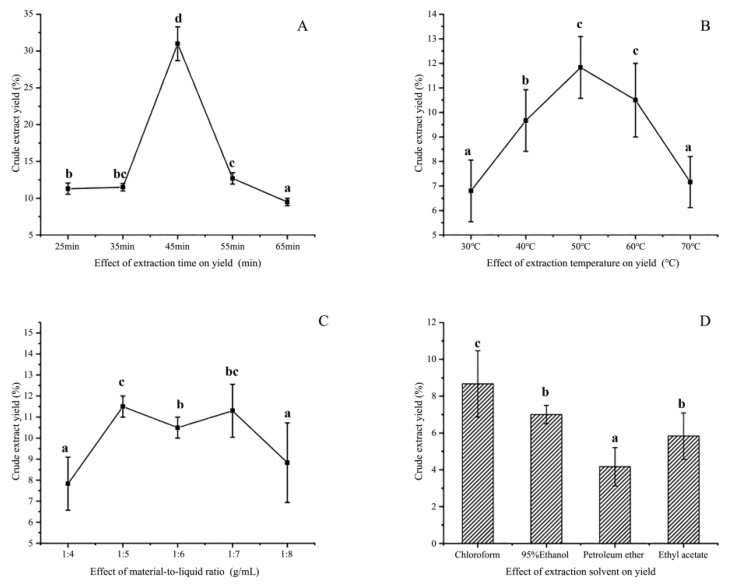
Effects of (**A**) the ultrasonic extraction time, (**B**) extraction temperature, (**C**) material-to-liquid ratio, and (**D**) extraction solvent on the Cers yield (%). Varying lowercase letters (a–d) indicate statistically significant differences (*p* < 0.05).

The changes in various factors impacting the extraction of Cers yield from sea red rice bran are shown (Figure 1).

#### 3.1.1. Extraction Solvent

Different solvents caused varying degrees of damage and erosion to the cell walls of rice bran (Figure 1D). First, a fixed value was selected, and the four solvents were subjected to the same conditions of 45 min ultrasonic time and 360 w fixed ultrasonic power. The effects of the four solvents on the yield of Cers were studied at a material-to-liquid ratio of 5 g/mL and an extraction temperature of 60 °C. 

Chloroform and 95% ethanol exerted a significant effect on the Cers yield (Figure 1). However, the multi-factor analysis indicated that chloroform was highly toxic, due to residual toxic effects exerted by it on the products as well as on the human body. Ethanol and water are generally considered safe and environmentally friendly alternatives. This suggested that 95% ethanol may be considered the best solvent [26].

#### 3.1.2. Extraction Time

The effect of ultrasonic extraction time on the Cers yield is shown (Figure 1A). Ultrasonic time was set to different extraction times of 25, 35, 45, 55, and 65 min, while the other parameter was set to a fixed ultrasonic power of 360 W. The ratio of material-to-liquid was 5 g/mL, and the extraction temperature was 50 °C. Cers production continued to increase within the 25–45 min range, reaching a critical point at approximately 45 min, and then declining rapidly (Figure 1A). This observation may be attributed to the ultrasonic treatment increasing the frequency of molecular collision in the reaction system within a certain range, thereby, accelerating the reaction process. Thus, excessive ultrasonic time may adversely affect the stability of the target product, resulting in a decline in yield. Consequently, the 45 min extraction time was most suited for the RSM design.

#### 3.1.3. Extraction Temperature

Temperature changes exerted a significant effect on Cers products. The ultrasonic time was set to 45 min, while the ultrasonication power was fixed at 360 W (Figure 1B). The material-to-liquid ratio was set to 5 g/mL, while the extraction temperature was varied (30, 40, 50, 60, and 70 °C). Cers yield gradually increased with the temperature until it reached a maximum at approximately 50 °C (Figure 1B). After 50 °C, the yield began to decline slowly, showing a slope-like decline after 60 °C, which could be mainly attributed to the influence of continuously high temperatures on the substance stability. These results suggested that the yield could be maximized only within a certain temperature range, and therefore 50 °C was selected as the optimal extraction temperature for RSM.

#### 3.1.4. Material-to-Liquid Ratio

Cers yield is affected by the material-to-liquid ratio, with different raw materials being characterized by different ratios. The effect of the ultrasonic extraction time on the Cers yield is shown (Figure 1C). The ultrasonication time was set to 45 min, and the ultrasonication power was fixed at 360 W, while the extraction temperature was set to 50 °C, and the material-to-liquid ratios were 1:4, 1:5, 1:6, 1:7; and 1:8. 

The material-to-liquid ratio demonstrated a rapid upward trend within the range of 4 to 5 g/mL, which then decreased slowly and again increased gradually within the range of 5 to 7 g/mL, the difference in change within this range being insignificant (Figure 1C). The decrease within the range of 7 to 8 g/mL was significant. These results indicated that a higher material-to-liquid ratio does not necessarily lead to better results but only to a lower density and viscosity, thereby, reducing the concentration of the extract. Increasing the feed liquid would also produce waste. Therefore, 5 g/mL was chosen as the optimal material-to-liquid ratio for RSM.

### 3.2. Optimised Design by RSM

#### 3.2.1. Model Fitting and Statistical Analysis

Using the Box–Behnken design principle, 17 groups of variables were analyzed under the following conditions: a single-factor experimental result extraction time of 45 min (A), an extraction temperature of 50 °C (B), and a material-to-liquid ratio of 5 g/mL (C) as experimental factors. The Cers yield was the response value (Table 1), and 95% ethanol was used as the extraction solvent. The aim of the design according to BBD was to determine the optimal ratio of the extracted variables. 

The experimental data conformed to the quadratic polynomial model shown (1). Design-Expert version 12.0.3 was used to fit a multiple regression model to the experimental data, which could be used to determine the coefficient values corresponding to target responses and to construct the final prediction equation in the form of a quadratic multiple regression equation (2) depicting the Cers yield (Y) versus the extraction time (A), extraction temperature (B), and material-to-liquid ratio (C):

(1) $${\;Y=\alpha }_{0}+\sum_{i=1}^{\;3}\alpha_{i}X_{i}+\sum_{i=1}^{\;3}\alpha_{\mathrm{ii}}X_{i}^{2}+\sum_{i=1}^{\;n3}\;\sum_{j=1}^{\;3}\alpha_{\mathrm{ij}}X_{i}X_{j}$$

where Y is the response variable (the Cers yield extract), α_0_ is the constant coefficient, α_i_ is the linear coefficient, α_ij_ is the interaction coefficient, α_ii_ is the square coefficient, X_i_ and X_j_ are the coding independent variables, and X_i_, X_j,_ and X_i2_ are the interaction and quadratic terms.

(2) $$Y=12.46+0.3375A+0.425B+0.6125C+0.275\mathrm{AB}+0.2\mathrm{AC}-0.025\mathrm{BC}-1.805A^{2}-2.13B^{2}-2.155C^{2}$$

The experimental values of these 17 experiments were input into Design-Expert software version 12.0.3 for model fitting and error calculation, the output of which is shown (Table 1). The ANOVA results of the Cers yield in sea red rice bran are also shown (Table 2).

The model (F = 271.08; *p* < 0.0001) indicated a significant difference (Table 3). The *p*-value for the mismatch term (0.053), was not statistically significant, indicating a small experimental error. The determination coefficients (R^2^ = 0.9971 and Adj-R^2^ = 0.9935) indicate 99.71% and 99.35% fitting degrees of the response values, respectively. The coefficient of variation, CV (%), which was 1.69, indicated that the model had good reproducibility. This showed that the experimental values obtained in this study had a high level of reliability and accuracy. 

In the regression model, F > 0.05 indicated that the model term was significant and that the influence of the model term was positively correlated with the F-value. Therefore, in this model, A, B, C, A^2^, B^2^, C^2^, AB, AC, and BC were significant model items, which exerted a degree of influence on the Cers yield. Furthermore, the *p* values of the primary terms, B and C, and the secondary terms, A^2^, B^2^, and C^2^, in the regression model were <0.0001, indicating that the terms were highly significant. 

**Figure 2:**
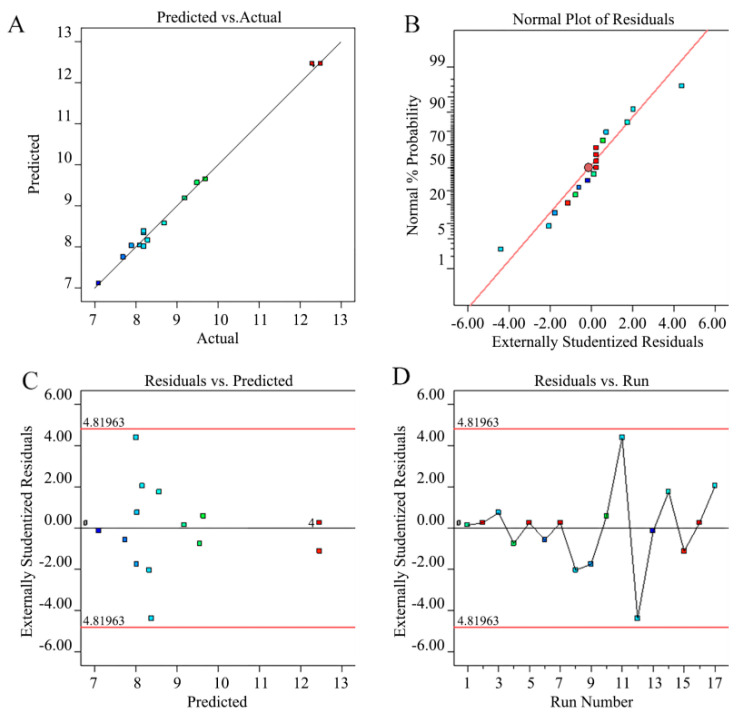
(**A**) Relationship between the predicted and test values, (**B**) normal error diagram of the RSM experiment value, (**C**) RSM residual and model prediction diagram, and (**D**) figure of residue versus the number of runs. The different colors represent the dispersion of the discrete random variables.

This shows that the association between the experimental factors and the response values was non-linear, rather than linear. The significance of the relationship of each factor was as follows: material-to-liquid ratio (c) > extraction temperature (b) > extraction time (a). The data shows that the predicted value agreed well with the actual values (Figure 2) and that the degree of fit between the model and the actual process was significant. 

The residual diagram of the RSM experiment was studied using Figure 2, which showed that the points of the normal residual diagram were almost on a straight line and that most were located in the mid-region. This showed that the predicted values were almost consistent with experimental values. Most discrete data points were concentrated in the middle of the straight line, indicating that the model was remarkably accurate and that most residual errors corresponding to the running numbers were close to 0 (Figure 2). A comprehensive analysis of the above data and charts indicated that the model could be used to effectively analyze and explain the influence of the material-to-liquid ratio, extraction temperature, and extraction time on the Cers yield [27].

#### 3.2.2. Response Surface Analysis

**Figure 3:**
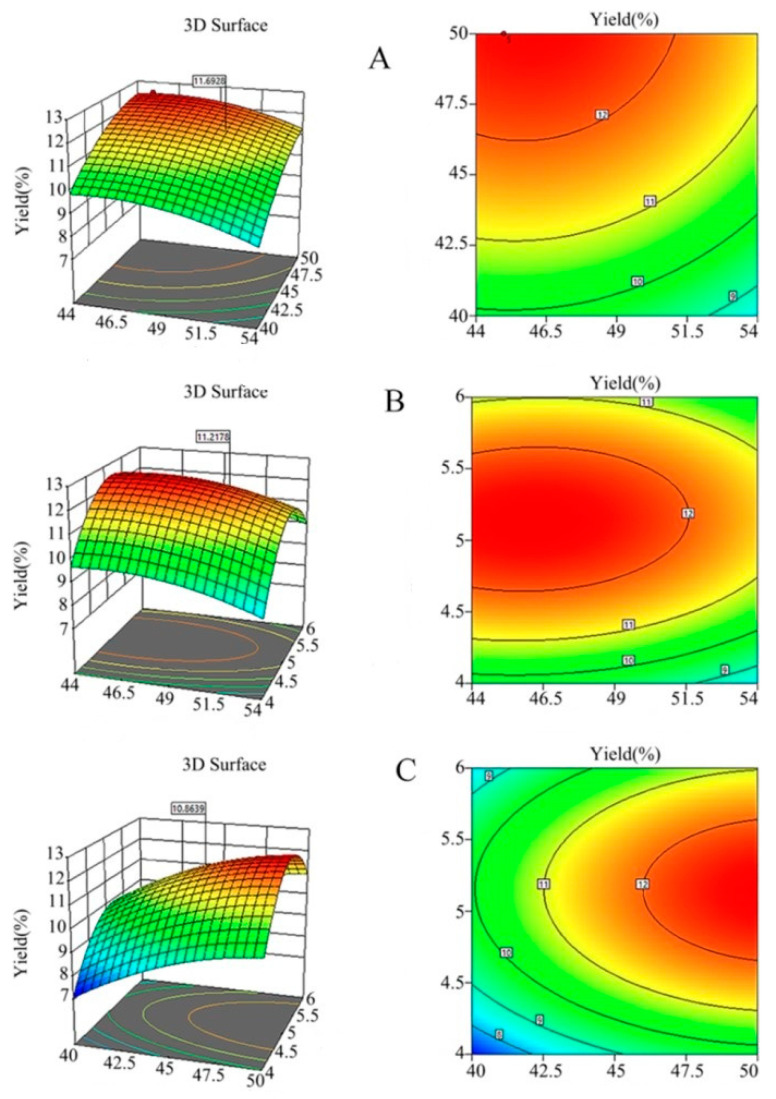
Effects of the (**A**) extraction time, (**B**) extraction temperature, and (**C**) material-to-liquid ratio on the Cers yield.

To provide a more detailed description of the interaction between dependent variables, a 3D response diagram was constructed. The shapes of the surface diagram and the contour diagram indicated the significance of the interaction between variables. The response surface model is shown (Figure 3).

The effects of the interaction between extraction time and temperature on the Cers yield under a fixed material-to-liquid ratio of 5 g/mL are shown (Figure 3A). The contour graph shows that the extraction time and temperature changed uniformly and that the contour lines are not dense. Thus, although the two factors showed some interaction, the overall effect on the Cers yield was not particularly significant.

The interaction between extraction temperature and material-to-liquid ratio on the Cers yield at 45 min extraction time is shown (Figure 3B). The contour line had a flat oval shape, indicating significant interaction between C and B. As the rice bran requires full and proper contact with the solid–liquid. The F-value of 0.094 and a *p*-value of 0.76 indicated that the Cers yield was significantly affected by the material-to-liquid ratio. The greater the F-value of the material-to-liquid ratio, the more significant the protrusion of the response surface. Here, the determining parameters were significantly large and the influence of temperature also accelerated the contact rate between the material-to-liquid ratio and material, thereby, causing the molecules to move violently.

The interaction between the extraction time and the material-to-liquid ratio on the Cers yield at an extraction temperature of 50 °C (F = 6.07; *p* = 0.04) is shown in Figure 3C. The material-to-liquid ratio and extraction time significantly affected the Cers yield, and as time progressed, the material-to-liquid ratio contacted the sample fully and enhanced the interaction. A shorter ultrasonication time may result in an insignificant optimization effect, as shown by the slope steepness, elliptical shape, and density of the contour line, indicating that the material-to-liquid ratio exerted a significantly large effect on neural extraction, thereby, highlighting the importance of exploring the interactions between the material-to-liquid ratio and extraction time [28].

### 3.3. Parameter Optimization and Model Verification

According to the optimization function of Design-Expert version 12.0.3, under the optimum conditions, the Cers yield from sea red rice bran reached a maximum value of 12.546%, with an optimum extraction time of 46.097 min, an optimum extraction temperature of 51.055 °C, and a material-to-liquid ratio of 5.146. The extraction process was then adjusted to an extraction time of 46 min, an extraction temperature of 51 °C, and a material-to-liquid ratio of 5 g/mL to reflect practical feasibility. Three groups of parallel experiments were conducted under the optimal conditions required for model optimization to verify the model predictability. Under these conditions, the average extraction efficiency of Cers crude extract was 12.1%—a value that was consistent with the prediction of the model.

### 3.4. Comparison of Different Extraction Methods

Traditional extraction techniques, such as SFE, as well as EAE and UAE, have been reportedly used for the extraction of Cers or sphingomyelins [29]. Cer yields obtained via traditional extraction techniques, SFE, EAE, and UAE, were 8.36 ± 0.03%, 10.48 ± 0.06%, and 12.48 ± 0.09%, respectively; these showed a statistically significant difference (*p* < 0.05). These results indicated that different extraction methods had exerted a significant effect on Cers extraction. For example, the low yield obtained via the traditional extraction method may be attributed to the hardness of the cell walls of rice bran, which prevents the extract from diffusing into the solvent. 

The yield obtained via EAE was slightly higher than that obtained via traditional solvent extraction, possibly due to the effect of cellulase on rice. The epidermis of the chaff cell wall shows varying responses to dissolution. However, as enzymes are largely affected by environmental factors, especially organic solvents and heat, reduced enzyme activity often becomes an issue [30]. By contrast, extraction via UAE was improved possibly due to the cavitation effect produced by ultrasound combined with organic solvents, which greatly promoted the degree of extract release [31]. 

Furthermore, the combined rate of the two properties was more significant. Thus, the extracted material showed improved biological activity with a higher degree of release. For example, although Cers types extracted via EAE were less than those extracted via UAE, the yields were similar. These results indicated that the yield, chemical properties, and biological activity of Cers may vary with the extraction techniques used, and these findings are consistent with those reported in the existing literature [32,33,34].

### 3.5. TLC and Cers Purification

**Figure 4:**
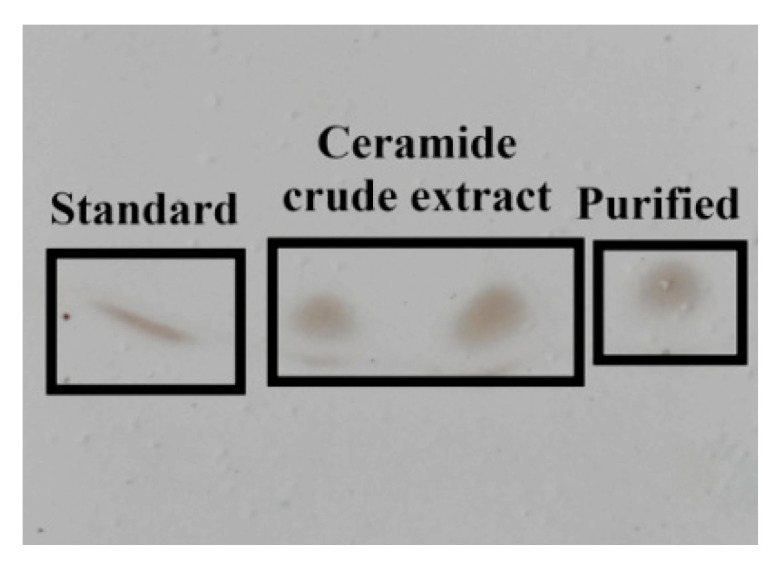
TLC chromatograms of sea red rice bran crude and purified extracts; a commercial rice bran Cer standard was used as a reference standard.

Cers reacts with copper sulfate solution under conditions involving phosphoric acid to generate active substances. When calcined at 130 °C, the generated macromolecular active substances form black spots on the thin plate due to carbonization, which was a unique outcome. The results showed that the R_f_ values of the Cer standard, crude Cer extract, and the purified product were essentially identical (Figure 4), and after measuring, their R_f_ value was about 0.57. Although the extract was dominated by sphingolipids, it also contained small amounts of phospholipids. 

As the extract contains varying types of Cer compounds, more carbides were found in the extracted samples, resulting in a higher degree of enrichment. Standard Cer contains a single type of compound with high purity (few impurities) and a low degree of enrichment [35]. The sequence of gradient elution for column chromatography on silica gel was numbered accordingly: eluent 1 (ethyl acetate:petroleum ether = 6:4); eluent 2 (ethyl acetate:petroleum ether = 1:1); and eluent 3 (acetone:petroleum ether = 6:4). Fractionation was performed by checking the elution position of Cer by TLC, and the Cer-rich fraction was then separated from other lipids, such as sterols, triacylglycerols, and sterol esters. Cers were collected from the final gradient identified by TLC. We were unable to identify and quantify lipid species using thin-layer chromatography. 

Therefore, these fractions were further characterized using UHPLC-Triple-TOF-MS/MS-based on the identity and content of various Cer molecules with closely related chemical structures. The Cers extract was purified via a silica gel column, separated, and purified thrice; following which, the eluent was collected, and white crystals were obtained.

### 3.6. Structural Characterization of Cers by UHPLC-Triple-TOF-MS/MS

Purified Cers from sea red rice bran were analyzed using high-speed mass spectrometry in negative ion mode to identify the types and contents of Cers. To identify Cer compounds, the generated molecular formulae and some source fragments were checked and studied, by comparing against different databases, such as PubChem, Phenol-Explorer, and the literature. According to the literature, Cers in plants do not exist in a single form, and instead, often exist as multiple linked complexes. The LC-MS method showed high selectivity and sensitivity in identifying Cers, thus, making it a powerful tool for analyzing complex sphingomyelins in plants [36]. LC combined with MS using electrospray ionization (ESI) and APCI sources has been widely used for identifying plant sphingosines [37]. 

Cers in sea red rice bran were screened using UHPLC-Triple-TOF-MS/MS. The identification of Cers is different from other methods of identification and analysis of polyphenols or flavonoids because most of the Cers are isomers, and thus one-to-one detailed analysis is not required.

**Figure 5:**
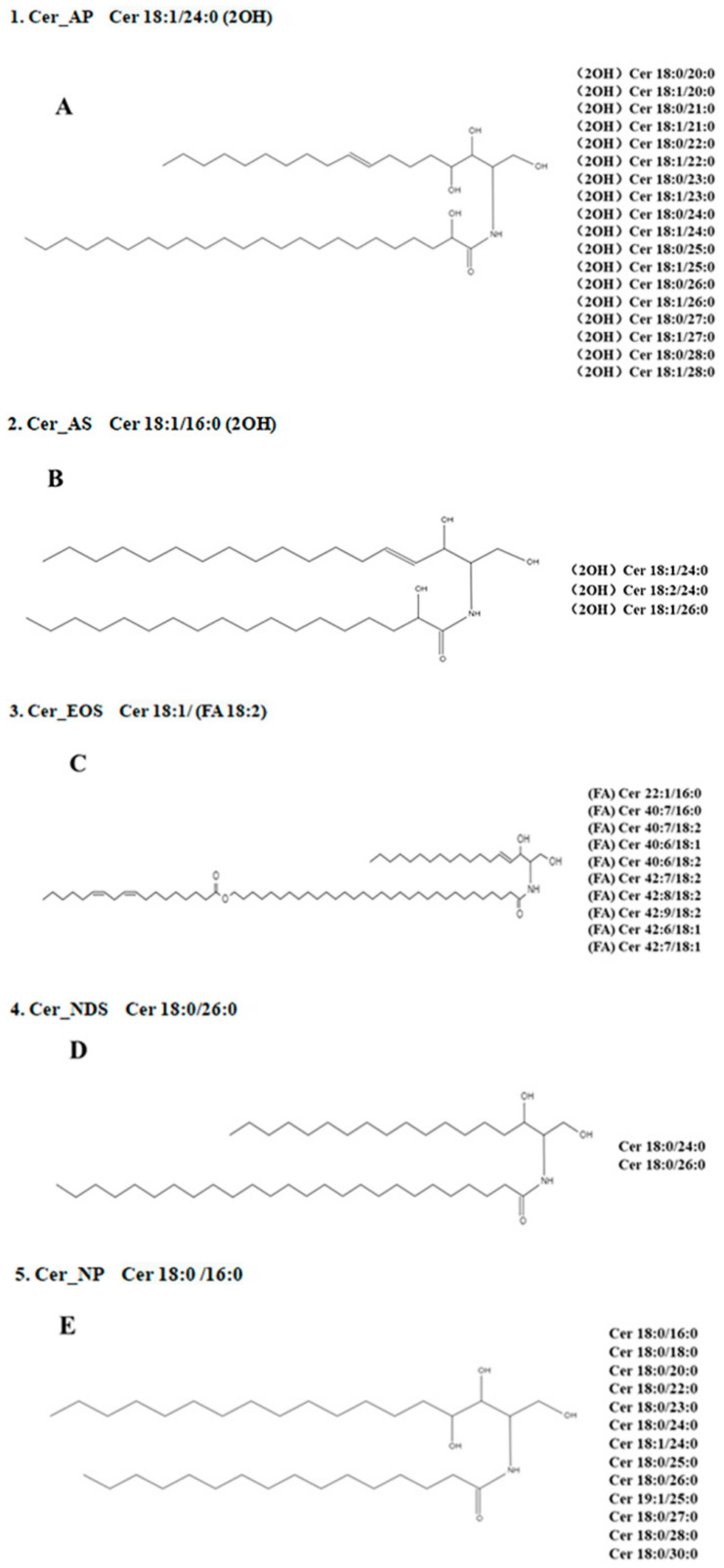
Structures of unique Cers in sea red rice bran (main category). (**A**–**E**) is the most important five types of Cers in sea red rice bran. N and EO indicate amide-linked FA species: N, non-hydroxy FA; A, a-hydroxy FA; and EO, omega-O-esterified FA. S, DS, and P indicate sphingoid bases: S, sphingosine; DS, dihydro-sphingosine; and P, phyto-sphingosine.

**Figure 6:**
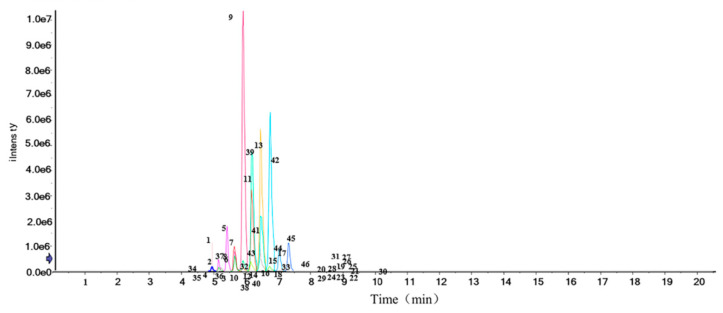
Ion chromatogram for the identification of total Cers in sea red rice bran by UHPLC-Triple-TOF-MS/MS.

In sea red rice bran, the following Cers were identified via a procedure similar to that used to identify phytoceramides. The identified Cer species and their structures are shown (Table 4 and Figure 5 and Figure 6, respectively). The loss was calculated and analyzed via mass spectrometry. The separation of Cer species using UHPLC was based on differences in the OH^−^, H^+^, and O^2−^ structures of carbon chains. During the MS analysis, all Cer species in sea red rice bran were identified as [M+H]—with a mass accuracy < 2 ppm for all cases. Quantitative data were referenced against a Cer synthetic internal standard containing Cer d18:1/15:0 (Cer_NDS). 

The internal standard Cer method was used to orient and calculate the accuracy. The selective screening was performed with an injection volume of 1 µL per sample. Quantification involved directly correlating the peak area generated by the internal standard with the peak areas of the discovered species. Specific differences between the sphingosine structures and their precursor ions were used to calculate and identify the remaining distinct Cer species, as well as the branched moieties of the identified Cers as hexose or hydroxyl groups [38,39]. 

Then, based on previous research on plant Cer peaks, the intensities of signals corresponding to molecular ions ([M+H]+) or fragment ions, ([M+H-H_2_O]+ and [M+H-Glc]+), during full scan MS, were used to further analyze the MS/MS structures. Since the properties of plant-derived Cers are relatively stable and include many types of sources, we aimed to understand the differences between the Cer types and contents contained in the bran of a new species of sea red rice. In this paper, we use the classification according to the Cer classification system introduced by Motta et al. [40]. In this paper we use the classification according to the CER classification system introduced by Motta et al. [33]

There were 46 kinds of Cer compounds in this sea red rice bran, resulting in an M/Z range of (Cer 18:1/20:0) 624.54907-[Cer 42:6; 3O (FA)] 918.78662). These 46 Cers were then divided into five major categories as follows: Cer_AP (cramide alpha-hydroxy fatty acid-phytospingosine); Cer_AS (ceramide alpha-hydroxy fatty acid-phytospingosine); Cer_EOS (ceramide esterified omega hydroxy fatty acid-sphingosine); Cer_NDS (ceramide non-hydroxyfatty acid-dihydrosphingosine); and Cer_NP (ceramide non-hydroxyfatty acid-phytospingosine). 

A total of five dominant Cer species were found in rice bran and identified as abundant ions based on a full-scan-based peak chromatogram. The analytical structures of these five Cers types are shown (Figure 5). The remaining Cer compounds were classified into five categories and used for analysis, among which, Cer_AP accounted for 18 species, Cer_AS for 3 species, Cer_EOS for 8 species, Cer_NDS for 2 species, and Cer_NP for 12 species. The time period was between 4.321 and 10.011 min, and Cer compound contents according to the peak areas ranged between 0.01% and 23.66%. The peak area size was related to the signal response degree of the substance. 

Furthermore, the larger the response value, the higher the peak area, and the larger the identical nature of the content and HPLC [41]. Among these, the contents of various Cer in Cer_AP were as follows: the Cer 18:0/24:0 (2OH) content was 23.66%, Cer 18:0/26:0 (2OH) was 14.91%, Cer 18:0/25:0 (2OH) was 7.32%, Cer 18:0/22:0 (2OH) was 2.81%, and Cer 18:1/24:0 (2OH) was 2.12%. Regarding the Cer contents in Cer_NP: Cer 18:0/24:0 was 11.96%, Cer 18:0/26:0 was 17.54%, Cer 18:0/25:0 was 6.10%, and Cer 18:0/28:0 was 2.51%, all of which were in sea red rice bran. 

The main Cer compounds, C_44_H_89_NO_4_ and C_42_H_85_NO_5_; and ion fragmentation at *m*/*z* = 682.63519 and 694.67273 produced the largest signal response, and multiple signal responses were identified nearby. Furthermore, ion fragmentation in these two also increased the richness of other fragments. The two signals represented the two main categories, Cer_AP and Cer_NP, in sea red rice bran, respectively. They correspond to the FA (fatty acid) and N (sphingosine base) ions of the same Cer molecule. They correspond to the FA (fatty acid) and N (sphingosine base) ions of the same ceramide molecule.

All categories showed different bond lengths and forms based on differences in OH^−^ and O^2−^. Among the similar sphingosine bases, Cer 40:6; 3O (FA) was related to α-hydroxy FAs. These bases were associated with most saturated and unsaturated FAs containing even and odd carbon atoms, which are also found in barley, wheat germ, corn, and soybeans [42,43]. Commercial rice bran has also been previously tested by the assays used. Since Cers in sea red rice bran had not been previously reported, its type and content remained unknown. Research indicates that the major Cer species contained in sea red rice bran were very similar to the Cers found in commercial rice bran. 

The contents of Cer 18:0/24:0 (2OH), Cer 18:0/26:0 (2OH), Cer 18:0/24:0, Cer 18:0/25:0, and Cer 18:0/26:0 were approximately 1.57–7.10-times higher than those in commercial rice bran. Sea red rice bran can be considered an interesting source of Cers in terms of the type and content of Cers. However, whether it can replace commercial rice bran for the large-scale extraction of Cers remains to be determined.

### 3.7. Cers Quantification Analysis

Cer compounds have complex structures, vary in type, and are expensive and thus cannot be compared one-to-one with standard products. Even identical Cers classes show different structures due to different hydroxyl groups (OH^−^, O^2−^, or FA) on their carbon chains, thus, requiring MS analysis. MS/MS analysis combined with UHPLC separation enables the identification of a large number of species [44,45]. We used the internal standard method and peak area size to analyze Cer compounds both qualitatively and quantitatively. 

MS-based quantification methods for specific sphingoid bases vary, and Cer internal standards, which are similar in structure, ionization, and fragmentation characteristics, allow for the better quantification of Cer categories, thereby, improving the accuracy. The Cer types and contents in sea red rice bran are shown in Table 4. Similar types of sphingosine bases were displayed in sea red rice bran as found in legumes, which are associated with most saturated and unsaturated hydroxyl groups and FAs containing even and odd carbon atoms. 

Studies have found that sea red rice bran plants contained a large number of Cers, of which (the main category) Cer_AP accounted for 55.75%, Cer_NP for 43.19%, Cer_NDS for 0.15%, Cer_AS for 0.14%, and Cer_EOS for 0.77%, indicating that Cer_AP and Cer_NP accounted for the largest portion as well as variations. These results were consistent with our expected results. Interestingly, some of these results showed that human skin Cers also possess structures that are similar to those of plant Cers, such as Cer_AP and Cer_NP, found in sea red rice bran. 

As Cer_AP and Cer_NP have a large number of hydroxyl groups, hydrogen bonds can be established to increase the stability of their lipid phase, and if these Cers are important for the formation of skin-specific Cers, then applications in skin care are possible. As a newly developed species, it may be used as an alternative commercial source of specific Cers [46]. 

### 3.8. Antioxidant Activity Analysis In Vitro

**Figure 7:**
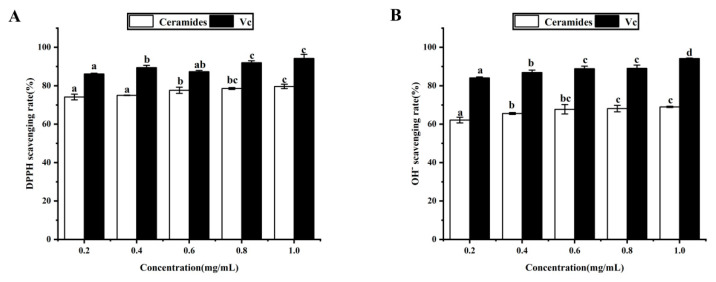
Effects of Cers on (**A**) DPPH and (**B**) hydroxyl free radical scavenging activity. Varying lowercase letters (a–d) indicate statistically significant differences (*p* < 0.05). Vc indicates the aqueous solution of the vitamin C standard used as a positive control.

In this study, we determined the antioxidant activity of Cers via their DPPH radical and hydroxyl radical scavenging activities. As the concentration was gradually lowered via dilution, the DPPH radical scavenging rates of Vc and Cer purified products remained within the 94% and 79% ranges, respectively, with the altered length showing that DPPH radical scavenging assay demonstrated good antioxidant activity (Figure 7 and Table 5). 

As the concentrations gradually decreased, the hydroxyl radical scavenging rates of Vc and Cer purified products remained within the 94% and 69% range, showing that the radical scavenging activity of Cers was dose-dependent. Compared with Vc, Cers showed a slightly weaker hydroxyl radical scavenging rate but a stronger increasing trend. This is because, due to the presence of hydroxyl (OH), Cers can reduce or inhibit the generation of free radicals by transferring hydrogen atoms. This is because of the presence of hydroxyl (OH), ceramides can reduce or inhibit the generation of free radicals by transferring hydrogen atoms [47,48]. 

### 3.9. Anti-Aging Activity of Purified Cers

Tyrosinase and elastase are enzymes that play a key role in melanin biosynthesis and skin aging in humans. They catalyze aerobic oxidation reactions in human skin that cause melanin accumulation and skin folds and accelerate skin aging. To evaluate the possibility of using Cers as anti-aging products, we assessed the efficacy of purified Cers for inhibiting the activities of tyrosinase and elastase. 

**Figure 8:**
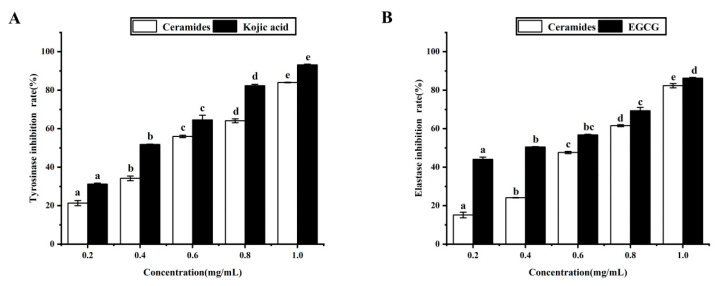
Effects of Cers on (**A**) tyrosinase inhibition and (**B**) elastase inhibition. Varying lowercase letters (a–d) indicate statistically significant differences (*p* < 0.05).

The results are presented (Figure 8 and Table 6). The percentage inhibitions of tyrosinase and elastase ranged from 21.31–84.01%, and 15.14–82.37%, respectively. Purified Cers exerted dose-dependent inhibitory effects on elastase and tyrosinase within a range of 0.2–1.0 mg/mL [49,50]. This indicated that Cers in sea red rice bran may be used to inhibit the activities of tyrosinase and elastase for skin whitening.

Cers are recognized for their skin-friendliness and are therefore commonly used as skin antioxidants and moisturizing agents. A recent study revealed that Cer molecules play an important role in establishing and maintaining skin barrier function [46]. However, the antioxidant and anti-aging capacity of Cers is influenced by various factors, including the structural composition, structural conformation, and molecular weight [51,52]. The efficacies of Cers from different plant sources were also found to differ during antioxidant and anti-aging capability testing. 

Reportedly, the performance of Cers extracted from poaceae in antioxidant and anti-aging tests was far superior compared to those extracted from legumes, and the survey also showed that far more types of Cers were extracted from poaceae than from legumes, indicating that the antioxidant and anti-aging activities of Cers were likely a result of the joint action of many different Cer configurations. Therefore, this factor should be taken into consideration when using Cers as raw materials for cosmetic purposes. The results of the study showed that Cers extracted from sea red rice bran exhibited good antioxidant and anti-aging activities during testing. 

## 4. Conclusions

In this study, Cer compounds were extracted and isolated from sea red rice bran via UAE and column chromatography purification. RSM analysis showed that the material-to-liquid ratio exerted the most significant effect on the Cers yield with the optimal yield of purified Cers obtained at 12.546%. Sea red rice bran extract was identified using thin-layer chromatography (TLC) and then purified and separated using silica gel column chromatography. Next, UHPLC-Triple-TOF-MS/MS was used to determine the Cer types that were present as well as their contents. 

As a result, 46 different types of Cers were identified in sea red rice bran, of which Cer 18:0/24:0 (2OH), Cer 18:0/26:0, Cer 18:0/26:0 (2OH), Cer 18:0/24:0, and Cer 18:0/25:0 accounted for 23.66%, 17.54%, 14.91%, 11.96%, and 7.32%, respectively, as the primary proportions. The antioxidant and anti-aging activities of the extracted Cer compounds were tested, and the results showed that Cers exhibited free radical scavenging and also inhibited tyrosinase and elastase activity. Therefore, these results suggest that Cers extracted from sea red rice bran may have potential value in the pharmaceutical and cosmetic fields.

## Figures and Tables

**Table 1:** Independent variables used in the Box–Behnken design and their level values.

			Level
**Variable**	−1	0	1
**Extraction time (min)**	35	45	55
**Extraction temperature (°C)**	40	50	60
**Material-to-liquid ratio (g/mL)**	1:4	1:5	1:6

**Table 2:** The Cers yield design matrix and response values.

Test Number	Extraction Time A	Extraction Temperature B	Material-to-Liquid Ratio C	Yield
1	0	1	1	9.2
2	0	0	0	12.5
3	−1	−1	0	8.1
4	1	1	0	9.5
5	0	0	0	12.5
6	−1	0	−1	7.7
7	0	0	0	12.5
8	−1	1	0	8.2
9	1	0	−1	7.9
10	1	0	1	9.7
11	0	1	−1	8.2
12	0	−1	1	8.2
13	0	−1	−1	7.1
14	−1	0	1	8.7
15	0	0	0	12.3
16	0	0	0	12.5
17	1	−1	0	8.3

**Table 3:** Variance analysis of the Cers yield response surface quadratic model.

Source	Sum of Squares	Df	Mean Square	F-Value	*p*-Value	>F
Model	64.30	9	7.14	271.08	<0.0001	significant
A-Extraction time	0.9113	1	0.9113	34.57	0.0006	
B-Temperature	1.45	1	1.45	54.82	0.0001	
C-Material-to-liquid ratio	3.00	1	3.00	113.87	<0.0001	
AB	0.3025	1	0.3025	11.48	0.0116	
AC	0.1600	1	0.1600	6.07	0.0432	
BC	0.0025	1	0.0025	0.0949	0.7671	
A^2^	13.72	1	13.72	520.47	<0.0001	
B^2^	19.10	1	19.10	724.77	<0.0001	
C^2^	19.55	1	19.55	741.88	<0.0001	
Residual	0.1845	7	0.0264			
Lack of Fit	0.1525	3	0.0508	6.35	0.0530	not significant
Pure Error	0.0320	4	0.0080			
Cor Total	64.49	16				
R^2^	0.9971					
Adj-R^2^	0.9935					
Pred-R^2^	0.9614					
Adeq precision	42.9463					
C.V.%	1.69					
*p* < 0.01 or *p* < 0.05						

**Table 4:** UHPLC-Triple-TOF-MS/MS identification of Cers.

NO	Compound	Formula	Rt (min)	Type	Ontology	Area	Percentage (%)	Classification Name	MS *m*/*z*	MS/MS
1	Cer 18:0/20:0(2OH)	C_38_H_77_NO_5_	4.948	[M-H]-	Cer_AP	652,223	0.26	Ceramide alpha-hydroxy fatty acid-phytospingosine	626.57129	327.28967
2	Cer 18:1/20:0(2OH)	C_38_H_75_NO_5_	4.725	[M-H]-	Cer_AP	254,409	0.10	Ceramide alpha-hydroxy fatty acid-phytospingosine	624.54907	370.33636
3	Cer 18:0/21:0(2OH)	C_39_H_79_NO_5_	5.169	[M-H]-	Cer_AP	214,132	0.09	Ceramide alpha-hydroxy fatty acid-phytospingosine	640.58142	341.30014
4	Cer 18:1/21:0(2OH)	C_39_H_77_NO_5_	4.934	[M-H]-	Cer_AP	123,692	0.05	Ceramide alpha-hydroxy fatty acid-phytospingosine	638.56104	384.33710
5	Cer 18:0/22:0(2OH)	C_40_H_81_NO_5_	5.407	[M-H]-	Cer_AP	6,691,605	2.81	Ceramide alpha-hydroxy fatty acid-phytospingosine	654.60211	362.34216
6	Cer 18:1/22:0(2OH)	C_40_H_79_NO_5_	5.164	[M-H]-	Cer_AP	1,680,557	0.70	Ceramide alpha-hydroxy fatty acid-phytospingosine	652.58353	309.30984
7	Cer 18:0/23:0(2OH)	C_41_H_83_NO_5_	5.661	[M-H]-	Cer_AP	3,394,601	1.46	Ceramide alpha-hydroxy fatty acid-phytospingosine	668.62238	323.32379
8	Cer 18:1/23:0(2OH)	C_41_H_81_NO_5_	5.401	[M-H]-	Cer_AP	509,753	0.22	Ceramide alpha-hydroxy fatty acid-phytospingosine	666.59741	369.33353
9	Cer 18:0/24:0(2OH)	C_42_H_85_NO_5_	5.91	[M-H]-	Cer_AP	54,053,688	23.66	Ceramide alpha-hydroxy fatty acid-phytospingosine	682.63519	382.36456
10	Cer 18:1/24:0(2OH)	C_42_H_83_NO_5_	5.656	[M-H]-	Cer_AP	4,853,410	2.12	Ceramide alpha-hydroxy fatty acid-phytospingosine	680.62280	382.37015
11	Cer 18:0/25:0(2OH)	C_43_H_87_NO_5_	6.186	[M-H]-	Cer_AP	16,389,775	7.32	Ceramide alpha-hydroxy fatty acid-phytospingosine	696.65405	397.36826
12	Cer 18:1/25:0(2OH)	C_43_H_85_NO_5_	5.901	[M-H]-	Cer_AP	747,527	0.33	Ceramide alpha-hydroxy fatty acid-phytospingosine	694.63794	397.36511
13	Cer 18:0/26:0(2OH)	C_44_H_89_NO_5_	6.459	[M-H]-	Cer_AP	32,721,940	14.91	Ceramide alpha-hydroxy fatty acid-phytospingosine	710.66913	410.39723
14	Cer 18:1/26:0(2OH)	C_44_H_87_NO_5_	6.177	[M-H]-	Cer_AP	1,769,607	0.80	Ceramide alpha-hydroxy fatty acid-phytospingosine	708.64484	411.38281
15	Cer 18:0/27:0(2OH)	C_45_H_91_NO_5_	6.735	[M-H]-	Cer_AP	1,082,791	0.50	Ceramide alpha-hydroxy fatty acid-phytospingosine	724.67841	425.39743
16	Cer 18:1/27:0(2OH)	C_45_H_89_NO_5_	6.436	[M-H]-	Cer_AP	131,391	0.06	Ceramide alpha-hydroxy fatty acid-phytospingosine	722.66553	425.39972
17	Cer 18:0/28:0(2OH)	C_46_H_93_NO_5_	7.006	[M-H]-	Cer_AP	591,917	0.28	Ceramide alpha-hydroxy fatty acid-phytospingosine	738.69855	281.25009
18	Cer 18:1/28:0(2OH)	C_46_H_91_NO_5_	6.725	[M-H]-	Cer_AP	164,664	0.08	Ceramide alpha-hydroxy fatty acid-phytospingosine	736.67798	438.43096
19	Cer 18:1/24:0(2OH)	C_42_H_83_NO_4_	8.952	[M-H]-	Cer_AS	227,151	0.10	Ceramide alpha-hydroxy fatty acid-sphingosine	664.62946	438.39212
20	Cer 18:2/24:0(2OH)	C_42_H_81_NO_4_	8.333	[M-H]-	Cer_AS	31,566	0.01	Ceramide alpha-hydroxy fatty acid-sphingosine	662.60376	438.3928
21	Cer 18:1/26:0(2OH)	C_44_H_87_NO_4_	9.418	[M-H]-	Cer_AS	67,966	0.03	Ceramide alpha-hydroxy fatty acid-sphingosine	692.65619	466.42711
22	Cer 22:1/(FA 16:0)	C_56_H_99_NO_5_	9.496	[M-H]-	Cer_EOS	34,843	0.02	Ceramide Esterified omega-hydroxy fatty acid-sphingosine	864.73761	255.2329
23	Cer 40:7/(FA 16:0)	C_56_H_97_NO_5_	9.063	[M-H]-	Cer_EOS	59,792	0.03	Ceramide Esterified omega-hydroxy fatty acid-sphingosine	862.72430	255.23362
24	Cer 40:7/(FA 18:2)	C_58_H_97_NO_5_	8.683	[M-H]-	Cer_EOS	107,728	0.06	Ceramide Esterified omega-hydroxy fatty acid-sphingosine	886.72754	279.23286
25	Cer 40:6/(FA 18:1)	C_58_H_101_NO_5_	9.535	[M-H]-	Cer_EOS	160,669	0.09	Ceramide Esterified omega-hydroxy fatty acid-sphingosine	890.75922	281.24467
26	Cer 40:6/(FA 18:2)	C_58_H_99_NO_5_	9.116	[M-H]-	Cer_EOS	202,413	0.12	Ceramide Esterified omega-hydroxy fatty acid-sphingosine	888.74298	251.23353
27	Cer 42:7/(FA 18:2)	C_60_H_101_NO_5_	9.168	[M-H]-	Cer_EOS	261,037	0.15	Ceramide Esterified omega-hydroxy fatty acid-sphingosine	914.75830	281.24771
28	Cer 42:8/(FA 18:2)	C_60_H_99_NO_5_	8.745	[M-H]-	Cer_EOS	191,375	0.11	Ceramide Esterified omega-hydroxy fatty acid-sphingosine	912.74392	279.23191
29	Cer 42:9/(FA 18:2)	C_60_H_97_NO_5_	8.302	[M-H]-	Cer_EOS	91,233	0.05	Ceramide Esterified omega-hydroxy fatty acid-sphingosine	910.72479	279.23110
30	Cer 42:6/(FA 18:1)	C_60_H_105_NO_5_	10.011	[M-H]-	Cer_EOS	26,952	0.02	Ceramide Esterified omega-hydroxy fatty acid-sphingosine	918.78662	281.24741
31	Cer 42:7/(FA 18:1)	C_60_H_103_NO_5_	9.588	[M-H]-	Cer_EOS	202,406	0.12	Ceramide Esterified omega-hydroxy fatty acid-sphingosine	916.77295	281.24913
32	Cer 18:0/24:0	C_42_H_85_NO_3_	6.709	[M-H]-	Cer_NDS	92,443	0.04	Ceramide non-hydroxyfatty acid-dihydrosphingosine	650.64062	292.38466
33	Cer 18:0/26:0	C_44_H_89_NO_3_	7.295	[M-H]-	Cer_NDS	91,370	0.11	Ceramide non-hydroxyfatty acid-dihydrosphingosine	678.67194	377.37521
34	Cer 18:0/16:0	C_34_H_69_NO_4_	4.321	[M-H]-	Cer_NP	152,341	0.05	Ceramide non-hydroxyfatty acid-phytospingosine	554.51074	256.23243
35	Cer 18:0/18:0	C_36_H_73_NO_4_	4.746	[M-H]-	Cer_NP	251,333	0.09	Ceramide non-hydroxyfatty acid-phytospingosine	582.52173	338.30356
36	Cer 18:0/20:0	C_38_H_77_NO_4_	5.162	[M-H]-	Cer_NP	791,603	0.31	Ceramide non-hydroxyfatty acid-phytospingosine	610.57343	352.31653
37	Cer 18:0/22:0	C_40_H_81_NO_4_	5.655	[M-H]-	Cer_NP	2,629,922	1.08	Ceramide non-hydroxyfatty acid-phytospingosine	638.60986	382.37323
38	Cer 18:0/23:0	C_41_H_83_NO_4_	5.914	[M-H]-	Cer_NP	2,184,516	0.91	Ceramide non-hydroxyfatty acid-phytospingosine	652.62201	353.33591
39	Cer 18:0/24:0	C_42_H_85_NO_4_	6.192	[M-H]-	Cer_NP	27,966,104	11.96	Ceramide non-hydroxyfatty acid-phytospingosine	666.64271	408.38483
40	Cer 18:1/24:0	C_42_H_83_NO_4_	5.925	[M-H]-	Cer_NP	327,691	0.14	Ceramide non-hydroxyfatty acid-phytospingosine	664.62523	408.3831
41	Cer 18:0/25:0	C_43_H_87_NO_4_	6.469	[M-H]-	Cer_NP	13,976,958	6.10	Ceramide non-hydroxyfatty acid-phytospingosine	680.65839	424.40680
42	Cer 18:0/26:0	C_44_H_89_NO_4_	6.751	[M-H]-	Cer_NP	39,370,848	17.54	Ceramide non-hydroxyfatty acid-phytospingosine	694.67273	450.43082
43	Cer 19:1/25:0	C_44_H_87_NO_4_	6.479	[M-H]-	Cer_NP	679,806	0.30	Ceramide non-hydroxyfatty acid-phytospingosine	692.65485	424.41461
44	Cer 18:0/27:0	C_45_H_91_NO_4_	7.034	[M-H]-	Cer_NP	4,450,536	2.02	Ceramide non-hydroxyfatty acid-phytospingosine	708.68988	464.44794
45	Cer 18:0/28:0	C_46_H_93_NO_4_	7.325	[M-H]-	Cer_NP	5,419,656	2.51	Ceramide non-hydroxyfatty acid-phytospingosine	722.70349	423.42868
46	Cer 18:0/30:0	C_48_H_97_NO_4_	7.896	[M-H]-	Cer_NP	375,501	0.18	Ceramide non-hydroxyfatty acid-phytospingosine	750.73138	494.49304
47	Cer d18:1/15:0	C_33_ H_67_NO_3_	4.336	[M-H]-	Cer_NDS	Internal standard	Ng	Ceramide non-hydroxyfatty acid-dihydrosphingosine	589.55314	369.55314

**Table 5:** Antioxidant activity of purified Cers.

Clearance Rate (%)	Samples	0.2 mg/mL	0.4 mg/mL	0.6 mg/mL	0.8 mg/mL	1.0 mg/mL
DPPH	Cers	74.14 ± 1.47 ^a^	75.00 ± 0.12 ^a^	77.63 ± 1.60 ^b^	78.56 ± 0.49 ^b,c^	79.65 ± 1.13 ^c^
	V_C_	86.12 ± 0.38 ^a^	89.45 ± 1.21 ^b^	87.36 ± 0.63 ^a,b^	92.01 ± 1.02 ^c^	94.23 ± 2.13 ^c^
OH^–^	Cers	62.13 ± 1.49 ^a^	65.56 ± 0.18 ^b^	67.77 ± 2.47 ^b,c^	68.13 ± 1.67 ^c^	69.01 ± 0.32 ^c^
	V_C_	84.10 ± 0.12 ^a^	86.93 ± 1.23 ^b^	88.89 ± 1.30 ^c^	89.06 ± 1.68 ^c^	92.18 ± 0.23 ^d^

Vc indicates the aqueous solution of the vitamin C standard used as a positive control. Varying lowercase letters (^a–d^) indicate statistically significant differences (*p* < 0.05).

**Table 6:** Anti-tyrosinase and anti-elastase activity of purified Cers.

Inhibition Rate (%)	Samples	0.2 mg/mL	0.4 mg/mL	0.6 mg/mL	0.8 mg/mL	1.0 mg/mL
Tyrosinase inhibition	Cers	21.31 ± 1.38 ^a^	34.20 ± 1.21 ^b^	56.0 ± 0.63 ^c^	64.12 ± 1.02 ^d^	84.01 ± 0.13 ^e^
	Kojic acid	31.23 ± 0.49 ^a^	51.81 ± 0.18 ^b^	64.54 ± 2.47 ^c^	82.36 ± 0.67 ^d^	93.21 ± 0.32 ^e^
Elastase inhibition	Cers	15.14 ± 1.47 ^a^	24.11 ± 0.12 ^b^	47.63 ± 0.60 ^c^	61.56 ± 0.49 ^d^	82.37 ± 1.13 ^e^
	EGCG	44.12 ± 1.12 ^a^	51.48 ± 0.23 ^b^	56.10 ± 0.30 ^b,c^	69.36 ± 1.68 ^c^	86.30 ± 0.33 ^d^

Varying lowercase letters (^a–e^) indicate statistically significant differences (*p* < 0.05).

## Data Availability

No new data were created or analyzed in this study. Data sharing is not applicable to this article.
